# Prostaglandin E_2_ As a Modulator of Viral Infections

**DOI:** 10.3389/fphys.2017.00089

**Published:** 2017-02-14

**Authors:** Willem J. Sander, Hester G. O'Neill, Carolina H. Pohl

**Affiliations:** Department of Microbial, Biochemical and Food Biotechnology, University of the Free StateBloemfontein, South Africa

**Keywords:** inflammation, prostaglandin E_2_, immunity, viral infection, therapeutic agents

## Abstract

Viral infections are a major cause of infectious diseases worldwide. Inflammation and the immune system are the major host defenses against these viral infection. Prostaglandin E_2_ (PGE_2_), an eicosanoid generated by cyclooxygenases, has been shown to modulate inflammation and the immune system by regulating the expression/concentration of cytokines. The effect of PGE_2_ on viral infection and replication is cell type- and virus-family-dependent. The host immune system can be modulated by PGE_2_, with regards to immunosuppression, inhibition of nitrogen oxide (NO) production, inhibition of interferon (IFN) and apoptotic pathways, and inhibition of viral receptor expression. Furthermore, PGE_2_ can play a role in viral infection directly by increasing the production and release of virions, inhibiting viral binding and replication, and/or stimulating viral gene expression. PGE_2_ may also have a regulatory role in the induction of autoimmunity and in signaling via Toll-like receptors. In this review the known effects of PGE_2_ on the pathogenesis of various infections caused by herpes simplex virus, rotavirus, influenza A virus and human immunodeficiency virus as well the therapeutic potential of PGE_2_ are discussed.

## Introduction

Viruses are small infectious agents that cause disease in all forms of life (Koonin et al., 2006). Based on their genomic material, they are classified as double-stranded (ds) DNA viruses, single-stranded (ss) DNA viruses, dsRNA viruses, (+)ssRNA viruses, (−)ssRNA viruses, ssRNA- reverse transcriptase (RT) viruses, and dsDNA-RT viruses (Baltimore, 1971).

To protect themselves from infection by viruses, hosts evolved immune systems (Janeway et al., 2004) consisting of many barriers and biological processes (Delves and Roitt, 2000). The immune system can be subdivided into innate immunity and adaptive immunity. The innate immunity is trigged when pathogens are identified by their pathogen associated molecular patterns (PAMPs) or when cells signal in response to damage, injury, or stress (Takeuchi and Akira, 2009). Although the innate system is non-specific and does not confer long-lasting protection (Mackay et al., 2000) it is the major defense mechanism against pathogens in most organisms (Litman et al., 2005). It consists of physical and chemical barriers including phagocytes and dendritic cells (DC), inflammation, the complement system, and natural killer cells. In contrast, the adaptive immunity relies on antigens and is highly specific to pathogens or pathogen-infected cells (Dörner and Radbruch, 2007). Lymphocytes are key role players in adaptive immunity and include both T cells and B cells (Janeway et al., 2004). Of the various components of the immune system the following are critical in clearance of viral infections; natural killer cells, interferons, dendritic cells, B cells, and T cells (Aoshi et al., 2011).

The immune system can be modulated by various factors including prostaglandins (PGs) (Harris et al., 2002). Prostaglandins are lipid molecules, derived from arachidonic acid (AA) and are produced by cyclooxygenase (COX), and PG synthases (Phipps et al., 1991). One of the most studied PGs is prostaglandin E_2_ (PGE_2_) which is produced by many cells including fibroblasts, macrophages and some malignant cells (Harris et al., 2002). PGE_2_ regulates various processes in the body via PGE_2_ receptors (EP1–EP4) (Sugimoto et al., 2000). Both the innate and adaptive immunity can also be regulated by the levels of PGE_2_, which can either have adverse or beneficial effects on the immune system's ability to fend off pathogens (Kalinski, 2012). This review focuses on the regulatory role of PGE_2_ on the immune system in the course of some well- viral infections caused by herpes simplex virus, Epstein-Barr virus, rotavirus, influenza A viruses, human immunodeficiency virus and hepatitis B virus, and the development of related potential therapies for the treatment of these infections.

## Production and function of PGE_2_

Prostaglandins are eicosanoids that are produced by nearly all mammalian cells (Park et al., 2006). They are not stored within cells but rather produced in response to specific trauma, signaling molecules or stimuli such as infections (Smith, 1989; Funk, 2001). PGE_2_ is the most abundant prostanoid (Serhan and Levy, 2003) in the mammalian body and under normal physiological conditions and plays a role in regulation of immune responses, blood pressure, gastrointestinal integrity, fertility (Ricciotti and FitzGerald, 2011), and inflammation (Davies et al., 1984).

### Biosynthesis of PGE_2_

#### Phospholipase A_2_, the rate-limiting enzyme in PGE_2_ synthesis

**Figure 1:**
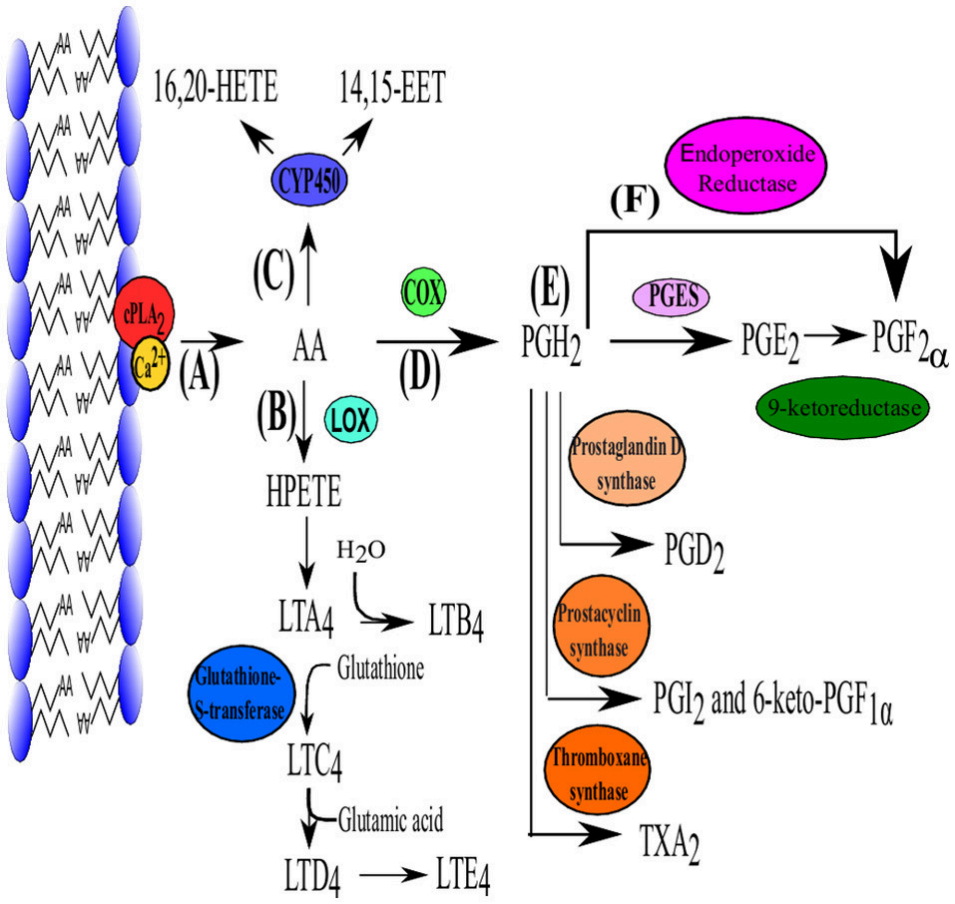
**The biosynthesis pathway of PGE_2_**. **(A)** Production of PGE2 is initiated with the liberation of AA by cPLA_2_. Arachidonic acid can then enter one of three pathways. **(B)** Lipoxygenase (LOX) converts AA to hydroperoxyeicosatetraenoic acid (HPETE) which is converted to leukotriene A_2_ (LTA_2_) and is further converted in the remainder of the leukotriene family (B_4_–E_4_) which are mainly responsible for lipid signaling. **(C)** Cytochrome P450 can also use AA as substrate which subsequently produces 16, 20- hydroxyicosatetraenoic acid (HETE) and 14, 15-epoxyeicosatrienoic acid (EET) which function in autocrine and paracrine signaling. **(D)** Arachidonic acid is converted to PGH_2_ by die COX isoenzymes. **(E)** Prostaglandin H_2_ is the precursor for all the other prostaglandins and can be converted into PGE_2_ (via PGE2 synthase [cPGES, mPGES-1 and mPGES-2)], PGD_2_ (PGD_2_ synthase), PGI_2_ (Prostacyclin synthase), TXA_2_ (TX synthase) which functions as a vasoconstrictor. **(F)** PG*F*_2α_ can be produced from PGH_2_ directly by endoproxide reductase or form PGE_2_ via 9-ketoreductase. Adapted from Jenkins et al. (2009).

PGE_2_ synthesis is initiated with the liberation of AA (a polyunsaturated fatty acid) from membrane phospholipids, by phospholipase A_2_ enzymes (PLA_2_) (Funk, 2001) (Figure 1A). Phospholipase A_2_ enzymes are divided into three major classes: Secreted PLA_2_ (sPLA_2_), intracellular group VI calcium-independent PLA_2_ (GVl iPLA_2_) and group IV cytosolic PLA_2_ (GIV cPLA_2_) (Murakami and Kudo, 2004). While all PLA_2_ enzymes can release AA from membrane phospholipids, only cPLA_2_α (a family member of GIV cPLA_2_) performs this reaction as a primary function (Leslie, 2004; Murakami and Kudo, 2004). Cytosolic phospholipase A_2_α has been found in most cells and tissues and is highly specific for the sn-2 bond of AA.

#### Production of PGH_2_, the precursor to prostanoids

Cyclooxygenases (Figure 1C) are membrane-bound heme-containing glycoproteins that have two major functions, namely the addition of a 15-hydroperoxy group to AA to form prostaglandin G_2_ (PGG_2_) and the reduction of the nascent hydroperoxy group of PGG_2_ to form prostaglandin H_2_. Cyclooxygenase has two isoforms COX-1 (constitutively expressed) and COX-2 (inducible). Although they are similar in structure and function, COX-2 utilizes endogenous AA while COX-1 uses AA derived from exogenous sources such as dietary intake (Park et al., 2006).

#### Prostaglandin E_2_ synthases

Prostaglandin H_2_(PGH_2_) is the substrate for prostaglandin E synthases (PGES) which produces the more stable prostanoid, PGE_2_ (Zurier, 2014) (Figure 1D) as well as for prostanoid synthases (Figure 1E). The production of PGE_2_ requires at least three PGESs, microsomal prostaglandin E synthase-1 (mPGES-1), mPGES-2 and cytosolic prostaglandin E synthase (cPGES) (Figure 1E). For a detailed review on PGES the reader is referred to Park et al. (2006).

#### Degradation

PGE_2_ is rapidly degraded *in vivo* by 15-hydroxyprostaglandin dehydrogenase and is therefore rapidly removed from tissues and circulation (Förstermann and Neufang, 1983; Tai et al., 2002).

### Prostaglandin E_2_ transport

Since PGs are produced intracellularly they need to be secreted to exert their extracellular effects (Park et al., 2006). The original prevailing notion was that newly synthesized PGs simply exited the cell via passive diffusion, as the electronegative interior of the cell favors the diffusion out of the cell (Schuster, 2002). However, the kinetics behind PG transport cannot be fully explained by this slow diffusion and a prostaglandin transporter (PGT) (Kanai et al., 1995) and multidrug resistance protein-4 (MRP4) (Reid et al., 2003) were found to import and export PGs, respectively. The prostaglandin transporter is a membrane spanning protein that is only expressed in prostanoid producing cells (Bao et al., 2002), while MRP4 is also a membrane spanning protein but is expressed in all cells (Russel et al., 2008).

### Prostaglandin receptors and signaling

**Figure 2:**
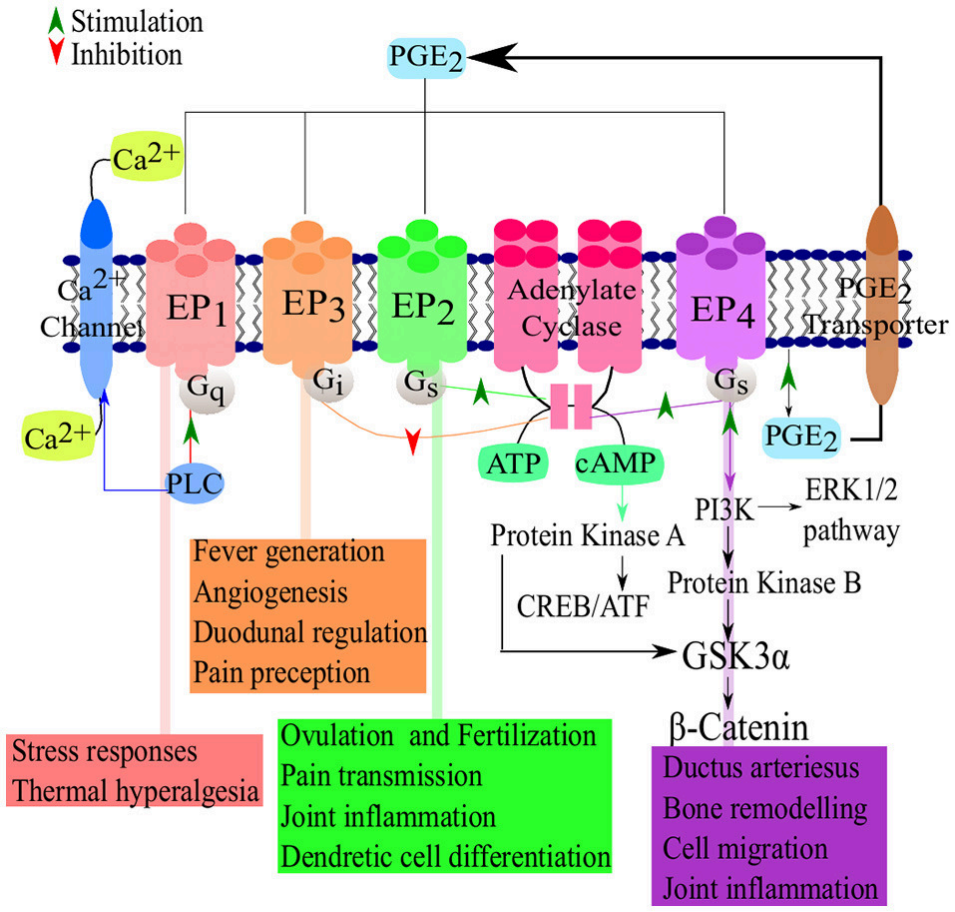
**PGE_2_-EP receptor signaling pathways**. Following the synthesis of PGE_2_, the prostanoid is exported and signals via four known receptors (EP1–EP4). The receptors then active cAMP/PKA/CREB signaling pathways which are responsible for the major suppressive and regulatory functions of PGE_2_. Adapted from Nasrallah et al. (2014) and Sugimoto and Narumiya (2007).

There are four PGE_2_ receptors, EP1, EP2, EP3, and EP4 (Figure 2). EP3 has several splice variants, adding an additional functional level to the receptor (Hata and Breyer, 2004). Of these four receptors, EP3 and EP4 have a higher affinity for PGE_2_ and thus require significantly lower concentration of PGE_2_ for effective signaling, compared to EP1 and EP2 (Kalinski, 2012). EP2 and EP4 mediate the anti-inflammatory and suppressive activity of PGE_2_ by signaling through G_*s*_-coupled receptors, mediated by the adenylate cyclase-triggered cAMP/ PKA/CREB pathway (Fujino et al., 2005). While EP2 and EP4 share the same function, they are triggered by different concentrations of PGE_2_, allowing EP2 to mediate PGE_2_ functions over a longer time period and at a later stage of inflammation while EP4 is rapidly desensitized (Nishigaki et al., 1996). Although EP2 and EP4 signal in a cAMP-dependent manner, both have been shown to activate the GSK3/β-catenin pathway (Fujino et al., 2002). EP4 is also capable of signaling via the ERK1/2 pathway (Fujino et al., 2003). The signaling cascades of the EP receptors leads to the production of cAMP or the mobilization of Ca^2+^ which in turn leads to inflammation, pain, immunoregulation, mitogenesis and cell injury. EP1 and EP3 are not dependent on G_s_- coupled receptors and lack any cAMP-activating functions. Instead, EP3 has cAMP-inhibiting functions. EP1 signals via Ca^2+^ release (Hata and Breyer, 2004) while most of the splice variants of EP3 signal via G_i_-coupled PGE_2_ receptors and some G_s_-coupled (Sugimoto et al., 1992). The differences between the various PGE_2_ receptors allow for adaptable patterns and responses of various cells types at certain stages in immunity (Kalinski, 2012). The reader is referred to Dennis and Norris (2015)for an in-depth review of eicosanoid signaling in infection and inflammation.

## Viral immunity

**Figure 3:**
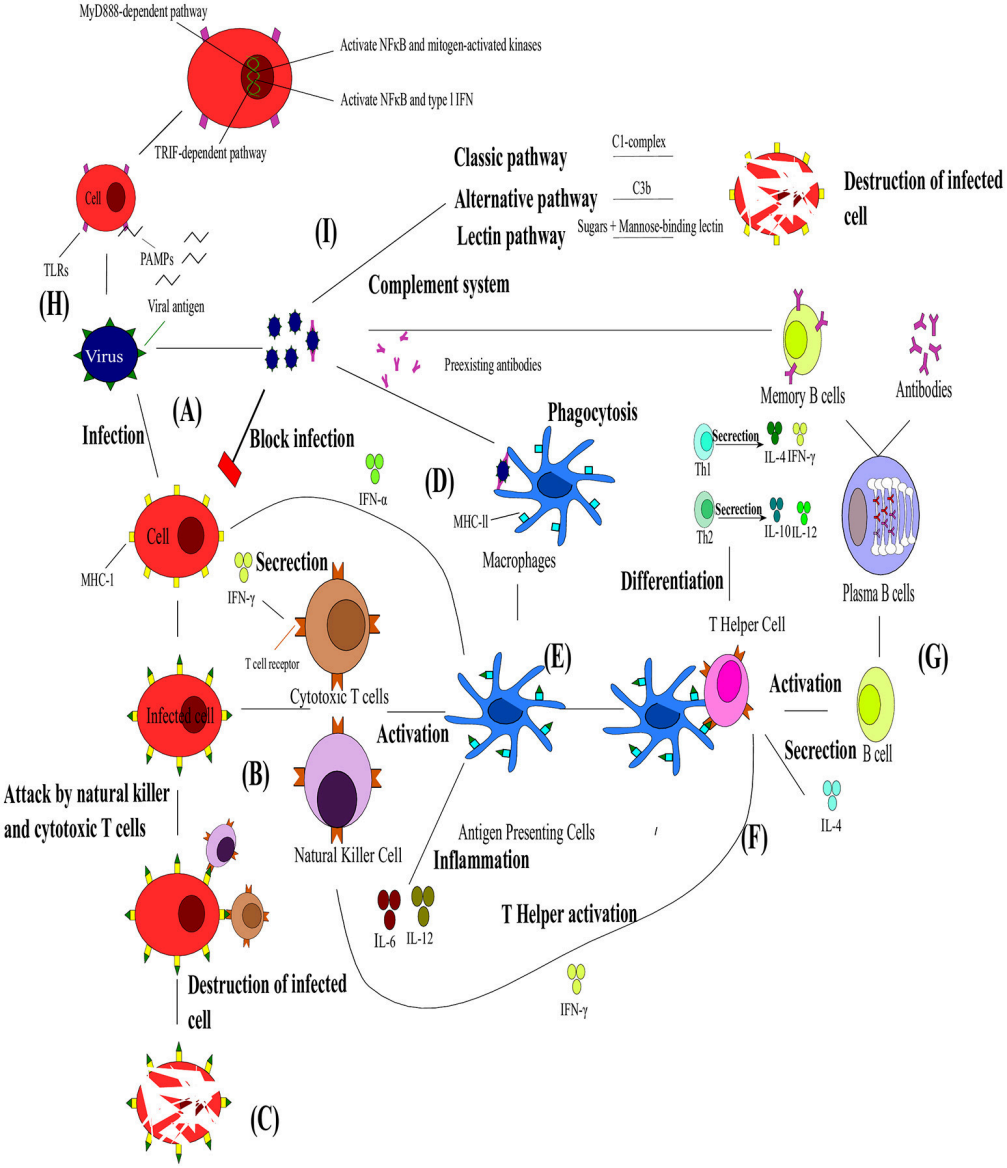
**The interaction between the innate and adaptive immunity in the presence of pathogens. (A)** Upon viral infection the infected cell presents the viral antigen on the major histocompatibility complex (MHC)-I. **(B)** Cytotoxic T cells (Tc) and natural killer cells (NK) can then bind to these viral antigens and **(C)** lead to the destruction of the cell. **(D)** Viral particles neutralized by pre-existing antibodies can be engulfed by macrophages via antibody neutralization. **(E)** This leads to viral antigens being presented by dendritic cells (DC), shown in blue on MHC-ll and the resulting antigen presenting cells (APC) activating Tc and NK and releasing cytokines. **(F)** T helper cells bind to these viral antigens and differentiate into Th1 or Th2 responses. T helper cells are also responsible for the activation of B cells. **(G)** The B cells transform into plasma cells which start producing antibodies specific toward the antigen and differentiate into B memory cells. **(H)** Toll-like receptors are an integral part of the innate immunity and function via two pathways activating NFκB, mitogen-activated kinases and type I IFN. **(I)**. The complement system composes of different pathways that lead to the destruction of infected cells. Adapted from Rouse and Sehrawat (2010).

The innate immune system is critical for pathogen recognition (Mackay et al., 2000) and functions as the first level of defense. In general the immune system relies upon leukocytes, antibodies, the complement system and cytokines to remove pathogens or toxins from the host (Janeway et al., 2004). Leukocytes are subdivided into different types, namely neutrophils, eosinophils, basophils, lymphocytes and monocytes which are distinguished by their physical and functional characteristics. Of particular importance to this review are the lymphocytes, which can be divided into B cells, T cells and natural killer cells, as well as monocytes which differentiate into macrophages in resident tissues. Antibodies are produced by plasma cells (differentiated B-cells) in response to specific antigens and bind to antigens. They bind to antigens, before the complexes are phagocytized. The complement system enhances the ability of antibodies and phagocytic cells to remove pathogens and damaged cells from an organism and promotes inflammation (Figure 3I). Although the complement system is considered to be part of the innate immune systems, it can be brought into action by the adaptive immune system. The complement system is known to consist of three components. These include, the classical pathway (relies on the activation of C1-complex by antibodies), the alternative pathway (relies on direct interaction between the pathogen and C3b) and the lectin pathway (relies on binding of certain sugars to mannose-binding lectin). Lastly, cytokines are a group of small molecules that are important in cell signaling and some as immunomodulatory agents. Cytokines play an important role in the balance between humoral and cell-based immunity.

Immune defenses against viruses are initiated when viral infection occurs or when antibodies from a previous infection recognize the virus and neutralize it (Mackay et al., 2000) (Figure 3A). Natural killer (NK) cells (Brandstadter and Yang, 2011) and interferons (IFN) are the most effective mechanism of the innate immunity against viral infections (Figures 3B,C). Natural killers cells are particularly important for the defense against the herpesvirus, poxvirus, papillomavirus, cytomegalovirus, influenza A virus, and human immunodeficiency virus (HIV) (Biron et al., 1989; Orange, 2002; Jost and Altfeld, 2013). These cells function mainly by killing infected cells by releasing cytotoxins early in infection, preventing spread to neighboring cells (Vivier et al., 2008). They also play a critical immunoregulatory role during the development of adaptive immunity. Thus, NK cell-mediated immunity plays a role in the control and clearance of viruses in the early stages of infection, but the continual stimulation of NK cells can contribute to viral pathology (Jost and Altfeld, 2013). However, there is still limited knowledge about the mechanisms of NK recognition and antiviral function. Another important response of the innate immunity toward viral infection is IFN (Samuel, 2001). Interferons are produced when a virus infects a cell (Sonnenfeld and Merigan, 1979). They are secreted as cell surface receptors causing the transcription of multitudes of IFN-stimulated genes which encode for protein products with antiviral, antimicrobial, antitumor and immunomodulatory effects (Fensterl and Sen, 2009). Type I (IFN-α, -β, -ω, -ϵ, -κ, -τ, -δ, -ν, and -ζ) and type III (IFN-λ) IFNs are induced when any cell recognizes PAMPs whereas type II (IFN-γ) is induced by other cytokines like IL-12 and expression is restricted to T cells and NK cells (Samuel, 2001; Fensterl and Sen, 2009). Viruses have also acquired various mechanisms to circumvent these actions of the innate immunity (Cerwenka and Lanier, 2001; Katze et al., 2002).

Another critical role player in innate immunity is a class of proteins called Toll-like receptors (TLRs) (Figure 3H; Xagorari and Chlichlia, 2008). Toll-like receptors are single, membrane spanning, non-catalytic receptors that are mainly expressed by macrophages and DCs. They are responsible for recognizing and responding to the PAMPs, leading to the activation of intracellular signaling pathways and altered gene expression. In turn, this allows the host immune system to detect pathogens and respond to their stimuli (Kawai and Akira, 2005). The signaling pathways that are activated by TLRs are responsible for the production of type I IFN, inflammatory cytokines and chemokines as well as the induction of immune responses responsible for eliminating pathogens (Kawai and Akira, 2006). Toll-like receptors signal via two distinct pathways, the MyD888-dependent and the TRIF-dependent pathways (Kawai and Akira, 2010). The MyD888-dependent pathway's primary effect is to activate nuclear factor kappa B (NFκB) and mitogen-activated proteins kinases, while activation of the TRIF-dependent pathway leads to the production of type I IFN and the transcription of NFκB. Sensing via TLRs is also responsible for the induction of DC maturation, which in turn initiates adaptive immune responses (Pasare and Medzhitov, 2004).

Dendritic cells and their system of antigen-presenting cells (APC) (Figures 3D,E) are another important component of immunity as they bridge the innate and adaptive immunity (Banchereau and Steinman, 1998; Chan et al., 2006). The main function of DCs are to activate naïve T cells by presenting processed antigen material on their surface (Banchereau and Steinman, 1998). However, before DCs can present the antigenic material, they have to complete a maturation cycle which is induced by either direct contact with pathogens or by interactions with other innate immune cells. Invading pathogens are sensed via extensive PAMP receptors which control the secretion of cytokines, migration, proliferation, and expression of major histocompatibility complex (MHC) II and co-stimulatory molecules in DCs (Steinman, 2003). The activation of DCs can be enhanced by activated NK cells (Chan et al., 2006).

The adaptive immunity's B and T cells are stimulated by DCs (Hess et al., 2016) (Figure 3G). B cells, which are the precursors to antibody secreting cells, directly recognize antigens through their B-cell receptors, while T cells are dependent on APCs for antigen recognition (Banchereau and Steinman, 1998). In viral infections, B cells (part of humoral immunity) begin their maturation process when they come in contact with viral antigens within the lymphatic system (Dörner and Radbruch, 2007). Naïve B cells have membrane-bound antibodies that effectively bind the viral antigen. Immediately following its contact with a specific viral antigen the B cell divides to become B memory cells and/or plasma cells. B memory cells express the same membrane-bound antibody as original naïve B cells and this is essential for a faster immune response to the particular viral antigen in future infections. Plasma cells also produce the same antibody as the original B cell, but are secreted into the bloodstream and neutralize the viral pathogen. T cells (part of cell-mediated immunity) express T cell receptors (TCR) and either CD4^+^ or CD8^+^ receptors (Sant and McMichael, 2012). As stated earlier, T cells cannot recognize antigens without receptor molecules (either MHC I or MHC II) which are membrane-bound on APCs. T cells mature into T helper cells (T_H_), cytotoxic T cell (T_c_), and T regulatory cells (Tregs). T helper cells express CD4^+^ receptors and are responsible for activation of T_c_, B cells and other immune cells (Alberts et al., 2002) (Figure 3F). As mentioned earlier, T_H_ function by producing either Th1 or Th2 responses (Figure 3F). T helper cells 1 are produced when DCs secrete IL-2 and IFN-γ cytokines. These Th1 cells then secrete their own cytokines (IFN-γ and TNF-β) which stimulate recruitment of other lymphocytes to inflammation areas, induce B cell antibody switching and activate T_c_ (Romagnani, 1992). The Th1 responses are thus essential for the removal of intracellular pathogens. T helper cells 2 are produced when APC present antigens to TCR along with costimulatory molecule B7, IL-4, and IL-2. T helper cells 2 then secrete their own cytokines (IL-4, IL-5, and IL-13) which promotes IgE production, blocks IFN-γ receptors, recruits and activates basophil and eosinophil leukocytes (Romagnani, 1992). The Th2 response is thus responsible for the control of extracellular pathogens. Cytotoxic T cells remove pathogens and infected host cells and express CD8^+^ receptors, while Tregs express CD4^+^ and CD25^+^ and help in distinguishing of self from non-self-molecules (Alberts et al., 2002).

## The role of PGE_2_ in inflammation and immunity

All the classic signs of inflammation (swelling, redness, heat, and pain) can be attributed to PGE_2_ (Funk, 2001). PGE_2_ causes redness and edema (of skin) by augmenting arterial dilation and microvascular permeability, increasing the blood flow into inflamed tissues. The pain caused by PGE_2_ results from the action on peripheral sensory neurons and on central sites within the spinal cord and brain. In addition, the binding of PGE_2_ to one of its various receptors can regulate the functions of macrophages, dendritic cells (DCs) and T and B lymphocytes (Ricciotti and FitzGerald, 2011). These regulating functions of cell types can in turn lead to both pro- and anti-inflammatory effects.

**Figure 4:**
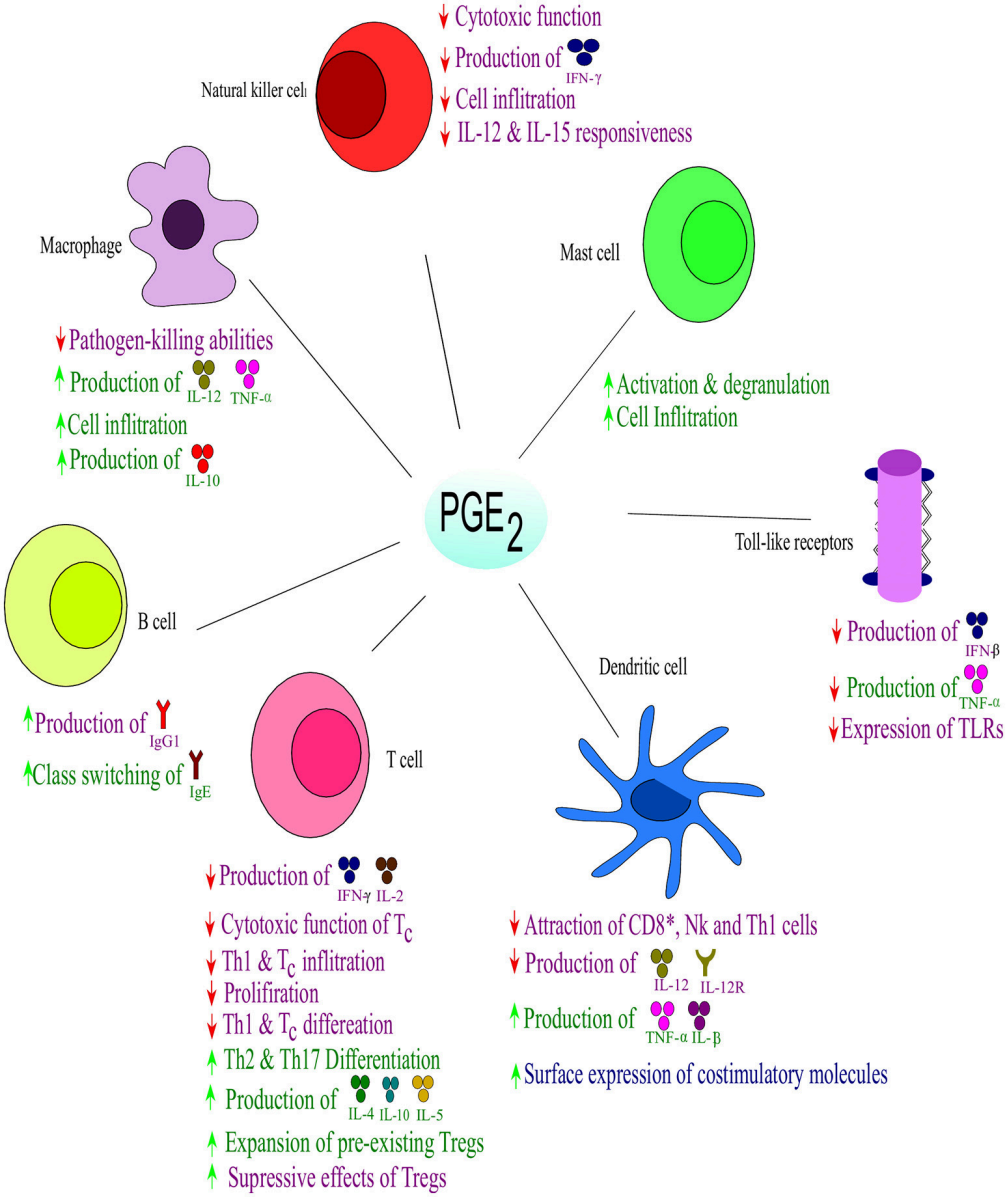
**Effect of PGE_2_ on immune responses**. Prostaglandin E_2_ suppresses the Th1- and natural killer (NK) cell-mediated type I form of immunity at their sites of induction, while supporting local acute inflammation and phagocyte mediated immunity. Prostaglandin E_2_ regulates the influx and activity of the effector vs. the regulatory cells into affected tissues. Purple indicates effects on immune suppression; blue indicates effects on immunity against intracellular pathogens, while green indicates effects on extracellular pathogens; ↑ increase; ↓ decrease. Interleukin (IL), interferon (IFN), tumor necrosis factor (TNF), Immunoglobulin (Ig). Toll-like receptors (TLRs) Adapted from Kalinski (2012).

PGE_2_ also plays a role in the regulation of cytokine expression in DCs and has shown bias in T cell differentiation toward either Th1 or Th2 responses (Kirkpatrick, 2005) (Figure 4). Disruption in the early stages of DC differentiation is noted as one of the effects of PGE_2_ on DCs (Kalinski et al., 1997). In addition to the suppressive function of PGE_2_ on differentiation of functionally competent Th1-inducing DCs, they also suppress responses of T_*c*_ (Obermajer et al., 2011). The induction of the DC migratory phenotype permitting their homing to drain lymph nodes, is enhanced by PGE_2_ (Kabashima et al., 2003; Legler et al., 2006). During early maturation, PGE_2_ can stimulate DCs to express co-stimulatory molecules which enhance T-cell activation (Krause et al., 2009). PGE_2_ can also enhance DC production of suppressive factors, but the net effect on DCs is to enhance promotion of T cell expansion (Kalinski, 2012). Dendritic cells that have matured in the presence of PGE_2_ have an impaired ability to induce Th1 while enhancing Th2 responses. PGE_2_ also has suppressive effects on naïve T cell activation and expansion as well as direct inhibitory effects on interleukin 12 (IL-12) production and the expression of IL-12 receptors (Kalinski, 2012). Furthermore, PGE_2_ also balances the Th cell responses by inhibiting interferon (IFN)-γ, a Th1 response. It does, however, not inhibit IL-4 and IL-5, Th2 responses, in CD4^+^ T cells (Snijdewint et al., 1993) (Figure 4). PGE_2_ is responsible for the suppression of IL-2 production and IL-2 responsiveness in T cells, leading to the suppression of T cell activation and proliferation at high doses (Kalinski, 2012). At lower doses PGE_2_ already shows a great modulatory effect on the shifting patterns of CD4^+^ T cell responses form the aggressive Th1 cells toward Th2 and Th17 cells that cause less tissue destruction. The Th1 suppressive effect of PGE_2_ also relies on the suppression of IL-12 in macrophages and DCs (van der Pouw Kraan, 1995; Kalinski, 2012). Thus, PGE_2_ shifts the immune response from Th1 to Th2, which leads to a reduced protective ability against intracellular pathogens (viruses and bacteria). In addition to the direct effect of PGE_2_ on Th1 immune cells, recent studies have showed the indirect effect of PGE_2_ in enhancing the development and activity of suppressive types of immune cells (Kalinski, 2012). PGE_2_ has been shown to promote the development of Tregs in both mice and humans (Baratelli et al., 2005). The EP2- and EP4-dependent induction of Tregs in murine cancer (Sharma et al., 2005) and skin UV irradiation (Soontrapa et al., 2011), have been shown to rely on COX-2 and PGE_2_. The Tregs have been shown to have with an role in human tumor tissues (Bergmann et al., 2007), The interaction between DCs and Tregs are also promoted by PGE_2_, suggesting a role in the promotion of the expansion of pre-existing Tregs (Muthuswamy et al., 2008). It has also been shown the PGE_2_ is involved in mediating the suppressive effect of Tregs (Mahic et al., 2006).

T helper cells 17 are important in the maintenance of mucosal barriers and the subsequent clearance of pathogens from these areas and have been implicated in autoimmune disorders and infectious diseases (Zambrano-Zaragoza et al., 2014). Cytokines such as IL-6, TGFβ, IL-21, and IL-23 are involved in the development of Th17 cells (Holzer et al., 2013). In addition, it has been shown that PGE_2_ plays a role in the differentiation and function of Th17 cells via cAMP and EP2/EP4 signaling (Boniface et al., 2009). Napolitani et al. (2009) concluded that PGE_2_ may dramatically influence the balance between the highly inflammatory IL-17 and the negative feedback loop exerted by IFN-γ. PGE_2_ accomplishes this by triggering the expression of EP2 and EP4 receptors on T cells, leading to a rapid increase in retinoic-acid-related orphan receptor-γt (controls T-cell precursors) and a decrease of T-cell specific T-box transcription factor 21 mRNA (encodes for a Th1 cell-specific transcription factor). The role Th17 plays in viral infection will be discussed in detail in a later section. Although no direct link has been found between PGE_2_ and autoimmune diseases, the role of Th17 cells have been reviewed in Zambrano-Zaragoza et al. (2014), implicating causative roles in Glioma, Multiple Sclerosis, Type 1 Diabetes and others. Since PGE_2_ can play a regulatory role in Th17 cell differentiation and functioning, it may be speculated to have some role in autoimmune diseases.

Toll like receptor 4 is well-known for its ability to recognize LPS and other ligands, including viral proteins, polysaccharides, and endogenous proteins (Brubaker et al., 2015). PGE_2_ has strong suppressive effects on LPS-induced IFN-β production at mRNA and protein levels in murine J774A.1 macrophages (Xu et al., 2008). This inhibitory effect is mediated through EP2 and EP4 which in turn activates the Epac/PI3K/Akt pathway. In contrast, PGE_2_ inhibits LPS-induced TNF-α by a PKA pathway independent of the Epac/PI3K/Akt pathway. Importantly, it was found that blocking COX activity *in vivo* resulted in higher concentrations of IFN-β post-LPS. Degraaf et al. (2014) showed that PGE_2_ can decrease the expression of TLR4. The authors showed that the effect was not by regulation of TLR4 mRNA but rather by the inhibition of its translation. They concluded that this reduction was mediated by EP2-dependent cAMP activated by PKA. The reduction in TLR4 expression was enough to decrease the transcription and section of TNF-α alveolar macrophages in responses to LPS. This thus shows that lipid mediators can modulate TLR4-mediated immune responses.

PGE_2_ can also interfere with early B cell activation and play a cAMP-mediated role in the regulation of immunoglobulin (Ig) class switching in B cells (Simkin et al., 1987; Phipps et al., 1991). Antibody class switching is a process where B cells change the production of antibodies from one type to another by a mechanism called class switch recombination (reviewed in Stavnezer and Amemiya, 2004). One of the most important consequences of these effects is the promotion of IgE production by PGE_2_ contributing to atopic disease (Carini et al., 1981).

Furthermore, PGE_2_ can also exert anti-inflammatory effects on neutrophils, macrophages, mast cells and NK cells which all form part of the innate immune system (Kalinski, 2012) (Figure 4). In NK cells, PGE_2_ functions by suppressing the responsiveness of IL-12 (Walker and Rotondo, 2004) and IL-15 (Joshi et al., 2001) which suppresses the cytolytic effects of NK cells (Bankhurst, 1982). PGE_2_ abrogates NK cell “helper” function by inhibiting the ability of NK cells to produce IFN-γ (Mailliard et al., 2005). The function of macrophages are modulated by PGE_2_ in an EP2-dependent (Aronoff et al., 2004) and phosphatase and tensin homolog-dependent manner (Canetti et al., 2007) which limits phagocytosis by alveolar macrophages and their pathogen-killing function (Serezani et al., 2007). This inhibition is in part mediated by induction of IL-1R–associated kinase-M, which blocks the scavenger receptor-mediated phagocytosis and the TLR-dependent activation of tumor necrosis factor-α (Hubbard et al., 2010). Furthermore, it has been shown that PGE_2_ in combination with LPS can promote anti-inflammatory phenotypes in macrophages by high expression of IL-10 and regulatory markers, SPHK1 and LIGHT, via a protein kinase A-dependent pathway (MacKenzie et al., 2013). The local attraction and degranulation of mast cells are induced by PGE_2_ in a mechanism involving EP1 and EP2 (Hu et al., 1995; Gomi et al., 2000).

Various steps of inflammation can thus be modulated by PGE_2_ in either a pro-inflammatory or anti-inflammatory manner. PGE_2_ can also modulate the immune system by shifting Th responses and thus affect the interplay between innate and adaptive immunity.

## Viral infection and PGE_2_

As reviewed above, PGE_2_ has varying effects on the immune system. In some instances viruses can interact with PGE_2_ and possibly benefit from the effects of PGE_2_ (Table 1). A few of the potential effects of PGE_2_ on various viral infections are described:

**Table 1:** **The effect of PGE_2_ on viral replication and infection**.

**Group**	**Virus**	**The effect of PGE_2_ on viral replication**	**Reference**
**(I) Double-stranded DNA viruses**	Herpes simplex virus	Increase viral replications	Harbour et al., 1978
	Cytomegalovirus	PGE_2_ contributes to immunosuppressive effect	Nokta et al., 1996
		PGE_2_ upregulation of major immediate promotor	Kline et al., 1998
		COX inhibitors decrease progeny virus but the effect is overcome by exogenous PGE_2_	Zhu et al., 2002
		PGE_2_ increase plaque formation and viral DNA copy numbers	Hooks et al., 2006
		PGE_2_ plays a role in direct cell-to-cell spreading	Schröer and Shenk, 2008
	Epstein Barr virus	Lytic reaction via EP signaling pathways	Gandhi et al., 2015
**(III) Double-stranded RNA viruses**	Rotavirus	COX inhibitors reduce duration of diarrhea	Yamashiro et al., 1989
		PGE_2_ might contribute to pathogenicity	Zijlstra et al., 1999
		PGE_2_ and COX-activity essential for Wa strain infection	Rossen et al., 2004
		Might be required for early infection i.e. attachment	Rossen et al., 2004
**(IV) (**+**) Single-stranded RNA viruses**	Coxsackie virus	Decrease viral titers	Xie et al., 2012
	Enterovirus 71	PGE_2_ might be required for replication	Tung et al., 2010a, 2011; Wang et al., 2015
	Sapovirus	PGE_2_ decreases the production of NO, leading to an increase in PSAV	Alfajaro et al., 2017
**(V) (**−**) Single-stranded RNA viruses**	Vesicular stomatitis virus	COX inhibitors/antagonist reduced viral production but the effect is overcome by exogenous PGE_2_	Chen et al., 2000
		COX-2 antagonist decreased viral titers	Chen et al., 2002
	Influenza A virus	PGE_2_ has an inhibitory effect on innate and adaptive immunity in mice	Liu et al., 2012
		PGE_2_ induces pro-inflammatory genes	Coulombe et al., 2014
		PGE_2_ activates expression of IL-27	Park et al., 2016
	Parainfluenza 3 virus	PGE_2_ inhibits viral replication	Luczak et al., 1975
	Lymphocytic choriomeningitis virus	PGE_2i_ ihibits the survival and effector functions of T_c_	Chen J. H. et al., 2015
	Respiratory syncytial virus	PGE_2_ causes a delayed protective RSV specific immune response	Bartz et al., 2002
		COX inhibitors reduced PGE_2_-dependent RNA transcription	Liu et al., 2005
**(VI) Single-stranded RNA-RT viruses**	Human T-lymphotropic virus type III	PGE2 causes an increased production of virus	Kuno et al., 1986
	Human immunodeficiency virus	PGE_2_ enhances HIV-1 long terminal repeat mediated reporter gene activation	Olivier and Tremblay, 1998
		PGE_2_ decreases virion penetration by suppressing expression of CCR5	Thivierge et al., 1998
		PGE_2_ inhibits virus replication by protein kinase A-dependent mechanism	Hayes et al., 2002
		PGE2 has an immunosuppressive effects when co-infected with HPV	Fitzgerald et al., 2012
		PGE_2_ reduces cell-to-cell spreading	Clemente et al., 2014
		PGE2 could play a role in pathogenicity via Th17 cell regulation	Zambrano-Zaragoza et al., 2014
**(VII) Double-stranded DNA-RT viruses**	Hepatitis B virus	PGE_2_ results in loss of viral replication	Flowers et al., 1994
		PGE_2_ decreases viral antigen	Hyman et al., 1999
		PGE2 could play a role in pathogenicity via Th17 cell regulation	Yang et al., 2013

### Double-stranded DNA viruses

#### Herpes simplex virus

Harbour et al. (1978) showed that when African green monkey kidney epithelial cells (Vero cells) were treated with PGE_2_ (0.1 to 10 μg/ml) for 24 h prior to infection by herpes simplex virus (HSV) type I (SC.I6 strain), there was a significant increase in the size of the plaques. When a low multiplicity of infection (MOI) (0.1) was used, PGE_2_ increased the viral yield, while no such effect was seen at high MOI (10). Furthermore, when Vero cells were treated with PGE_2_ inhibitors (mefenamic acid and indomethacin) there was a decrease in plaque size and inhibition of viral replication at a low MOI. This inhibitory effect can be overcome by the addition of exogenous PGE_2_. Taken together the results indicate that PGE_2_ does not increase viral production, but rather enhances the spread of the virus between cells. Work done by Thiry et al. (1985) on bovine herpes virus 1 (IBR/Cu5 strain) in Georgia bovine kidney cells (GBK cells) also showed an increase in the mean plaque size when GBK cells were treated with 0.1 and 10 μg/ml of PGE_2_. Treatment of HSV-tk (herpes simplex virus thymidine kinase)-transduced MC38 (murine colon cancer) cells with sulfasalazine (a NFκB inhibitor) lead to the inhibition of NF-_κ_B activity, inhibitor-κB phosphorylation and nuclear translocation of NF-_κ_B (Konson et al., 2006). This significantly decreased COX-2 expression and in turn reduced PGE_2_ release. In addition, these authors found that HSV-tk-transduced 9L (rat gliosarcoma) and T24 (human urinary bladder cancer) cells enhanced the expression of COX-2 and significantly increased PGE_2_ levels. When 4T1 (mouse mammary tumor cells) cells were infected with OSVP (a murine 15-PGDH inserted into the OSV viral genome) expression cassette) there was a significant decrease in PGE_2_ accumulation which led to the alleviation of immune suppression in mice (Walker et al., 2011). It can thus be concluded that PGE_2_ plays a role in HSV infection, although the exact mechanisms by which it facilitates viral replication and release are yet to be elucidated.

#### Cytomegalovirus

The infection of human T lymphocyte cells (MO cells) with cytomegalovirus (CMV) (AD169 strain) was found to induce the release of PGE_2_ via a TNF-α-dependent pathway (Nokta et al., 1996). This release of PGE_2_ apparently contributes to the immunosuppressive effects of CMV and could be involved in the pathogenesis of CMV. Kline et al. (1998) found that PGE_2_ can upregulate the major immediate promotor of human CMV (HCMV). They postulated that PGE_2_ could directly activate this promoter. Monocytoid cells (THP-1) were transfected with a plasmid containing the major immediate promoter gene and subsequently stimulated with PGE_2_. A synergistic increase in the promoter's activity was seen for PGE_2_ and other cytokines (IL-1β, TNF-α, IL-6, and IL-10). Human CMV may thus be activated by a mechanism which involves the activation of macrophages by these cytokines. The inhibition of COX-2 blocks the replication of HCMV, but when PGE_2_ is added exogenously the yield of infectious virus is substantially restored (Zhu et al., 2002). Hooks et al. (2006) showed that retinal pigment epithelial (RPE) cells infected with CMV induced COX-2 mRNA and protein synthesis increasing PGE_2_ levels. The increased levels of PGE_2_ enhanced CMV plaque formation and CMV DNA copy numbers. This indicates that PGE_2_ is required for the effective replication of HCMV, although the molecular mechanisms are yet to be elucidated. The treatment of CMV infected primary human foreskin fibroblast (HFF) with tolfenamic acid and indomethacin (inhibitors of COX) drastically reduced the direct cell-to-cell spread of CMV (Schröer and Shenk, 2008). The effect of these inhibitors is reversed by the addition of PGE_2_. This indicates that PGE_2_ is required for the effective replication of HCMV, although the molecular mechanisms are yet to be elucidated.

#### Epstein barr virus

Gandhi et al. (2015) investigated the link between chronic inflammation and the induction of Epstein Barr virus (EBV) lytic reactivation. They found that the addition of lipopolysaccharides (LPS) to cells latently infected with EBV, upregulated the expression of COX-2 and increased PGE_2_. The elevated levels of COX-2 and PGE_2_ coincided with gp350 (EBV late lytic protein) synthesis and detection of EBV in cell culture supernatant. When NS-398 (COX-2 inhibitor) was added to the cells, there was a drastic decrease in the levels of virus detected in cell culture supernatant. The overexpression of COX-2 and PGE_2_ in infected cells also coincided with the overexpression of PGE_2_ receptors EP1 and EP4. The addition of chemical inhibitors of EP1 and EP4 reduced the lytic reactivation of EBV even when COX-2 levels were upregulated. Taken together these results indicate that COX-2 is responsible for the lytic reactivation of EBV via PGE_2_ and its signaling via EP1 and EP4 receptor.

### Double-stranded RNA viruses

#### Rotavirus

Studies in infants found that there was an increase in PGE_2_ in both stool and plasma during rotavirus (RV) infection (Yamashiro et al., 1989). When these children were treated with a COX-inhibitor there was a reduction in the duration of diarrhea. Rotavirus infection also upregulates expression of both MHC I and MHC II in piglets (Zijlstra et al., 1999). Increases of CD8^+^ and CD4^+^ T-lymphocyte numbers were observed in the jejunum of piglets as well as elevated levels of PGE_2_. These observations suggested that PGE_2_ might contribute to RV pathogenicity. In 2009 Rodríguez and co-workers infected human Caucasian colon adenocarcinoma cells (Caco-2 cells) with RV and showed that the immunomodulators, IL-8, PGE_2_, and small quantities of TGF-β1, were released in RV infection Rodríguez et al. (2009). These immunomodulators are known to shift the T cell response to Th2 and may in part be responsible for the low number of T-cells in blood samples during RV infection. Infection of cells with RV is significantly inhibited when cells are treated with peroxisome proliferator-activated receptor gamma (PPARγ) and NSAIDs, indicating that RV has pro-inflammatory actions (Guererero et al., 2012). Moreover, PGE_2_ and COX-activity were shown to be essential for the establishment of RV (Wa strain) infection in Caco-2 cells (Rossen et al., 2004). Peroxisome proliferator-activated receptor gamma is responsible for the regulation of fatty acid storage and glucose metabolism. This inhibitory effect on RV indicates that fatty acids and their downstream products, like eicosanoids, may play a role in RV infection as it is well-known that RV associates with lipid droplets which is crucial for RV replication (Cheung et al., 2010). Rossen et al. (2004) postulated that PGE_2_ is necessary for early infection, most likely in viral protein synthesis and progeny production, rather than viral RNA production. The exact pathways behind these mechanisms remain unknown. It appears as if RV requires pro-inflammatory signaling for efficient replication, and that viral replication is inhibited by anti-inflammatory treatment (Guerrero and Acosta, 2016). There is a still a major gap in understanding the molecular mechanisms behind RV-induced inflammatory signaling.

### (+) Single-stranded RNA viruses

#### Coxsackie virus

Henke et al. (1992) showed that when human monocytes are infected with Coxsackie virus (B3 strain) (CVB3) they become activated and secrete cytokines. Higher levels of PGE_2_ and TNF-α were detected via ELISAs in infected cells compared to the uninfected cells. In 2011, Xie and co-workers showed that when anti-mouse IL-17antibody was added to block IL-17A (cardiac tumor-necrosis factor alpha) in BALB/c mice there was an increase in COX-2 proteins and PGE_2_ as well as a decrease in transforming growth factor beta (TGF-β) and TNF-α Xie et al. (2012). This was followed by a reduction in viral titers and decreased pathological scores. They also indicated that PGE_2_ and COX-2 play a role in viral myocarditis by being upregulated by IL-17A and TNF-α. The exact mechanistic role of these factors on CVB3 infection is yet to be elucidated.

#### Enterovirus 71

When rat brain astrocytic (RBA-1) cells are infected with enterovirus (strain 71) (EV71) they induce the expression of COX-2 and the subsequent release of PGE_2_ via c-Src/PDGFR/PI3K/Akt/p42/ p44 MAPK/c-Jun and NF-κB cascades (Tung et al., 2010b). Tung et al. (2010b) showed that human neuroblastoma (SK–N–SH) cells infected with EV71 also induced the expression of COX-2 and the production of PGE_2_ via a MAPKs (p42/p44 MAPK, p38 MAPK and JNK) and observed that the increase in PGE_2_ generation might be required for EV17 replication in infected cells. They postulated that EV17 replication by this COX-2/PGE_2_ mechanism may have an effect on the pathogenesis of central nervous system diseases. In 2011 Tung and co-workers found that the activation of the cAMP response element binding protein (CREB) in addition to the role of the c-Src/EGFR/p42/p44 MAPK signaling pathway in SK–N–SH cells were essential for EV71-induced COX-2 protein expression, COX-2 mRNA synthesis, and PGE_2_ production. They also found that PGE_2_ promoted further viral infection through EP2/EP4 receptors-cAMP signaling. Formononetin has been shown to reduce RNA and protein synthesis of EV71 in a dose dependent manner (Wang et al., 2015). This reduction was due to the inhibiting effects of formononetin on EV71-induced COX-2 expression and PGE_2_ production via the MAPKs pathway, including ERK, p38, and JNK.

#### Sapovirus

Alfajaro et al. (2017) showed that porcine kidney cells (LLC-PK) infected with porcine sapovirus (PSaV) Cowden strain, lead to an increase in the amount of COX-2 mRNA and protein levels while only transiently increasing the levels of COX-1. The authors also showed that the blocking of COX-1 and COX-2, by either NSAIDs or siRNAs, lead to a significant decrease in PGE_2_ and subsequently a decrease in the replication of PSaV. The viral proteins (VPg and ProPol) was found to be associated with activation of the COXs/PGE_2_ pathway. Furthermore, they observed that pharmacological inhibitors of COX-2 lead to a drastic increase in the production of NO, which lead to the reduction in PSaV replication that could be restored by inhibiting nitric oxide synthase via N-nitro-L-Methyl-Arginine-ester. The experimental data show that the sapovirus replication cycle depend or rely on the interaction with PGE_2_.

### (−) Single-stranded RNA viruses

#### Vesicular stomatitis virus

Chen et al. (2000) found that when Chinese Hamster Ovary (CHO) cells, infected with vesicular stomatitis virus (VSV) (Indiana serotype), were treated with aspirin, indomethacin (COX inhibitors) and celecoxib (COX-2 antagonist) there was an inhibitory effect on VSV propagation. They also found an increase in NO, a known inhibitor of VSV. Furthermore, when PGE_2_ was added to the cultures there was a significant decrease in NO and increase in viral yield. This indicates that the products of COX have an antagonistic effect on NO production which is in part responsible for the increase of VSV. Studies in mice (specific pathogen-free male BALB/c AnTac) by Chen et al. (2002) found that inhibition of COX-2 favors the Th1 response which lead to increased expression of nitric oxide synthase (NOS) -1 and that PGE_2_ also effects NOS. In addition they indicated that PGE_2_ might induce protein inhibitor of NOS (PIN) which binds to NOS-1 and prevents its activity. This indicates that PGE_2_ has a definite role in the propagation and infectivity of VSV, possibly through induction of PIN expression.

#### Influenza A virus

Influenza A virus (IAV) hyper induces the COX-2 and PGE_2_ production (Liu et al., 2012). The accumulation of PGE_2_ then activates a PKA-CREB signaling pathway, which in turn activates the IL-27/EB13 promotor, leading to the expression of IL-27. Interleukin-27 is responsible for the activation of signal transducer and activator of transcription 1 (STAT1) and STAT2 which inhibits viral replication (Owaki et al., 2008). Coulombe et al. (2014) showed that PGE_2_ has an inhibitory effect on both the innate and adaptive immunity when mice (C57BL/6, Taconic 4175, and Rag1^−/−^) were infected with IAV (A/Puerto Rico/8/34). PGE_2_ inhibited the recruitment and activities of macrophages via both EP2 and EP4. This had inhibitory actions on the type l IFNs and apoptosis pathways. The suppression of PGE_2_, via genetic ablation of mPGES-1 or by the pharmacological inhibition, improved survival after IAV infection, while the addition of PGE_2_ reversed this effect. They also showed that PGE_2_ inhibited T cell mediated immunity and thus concluded that IAV hijacks the mPGES-1/PGE_2_ pathway to suppress both innate and adaptive immunity in an IFN dependent manner. In 2016 Park and co-workers showed that the inhibition of mPGES-1 had anti-influenza effects by inhibiting PGE_2_ production and suppressing the induction of pro-inflammatory genes (TNF-α, IL-8, CCL5, and CXCL10). This anti-influenza effects was reversed by the addition of exogenous PGE_2_. The specific mechanism(s) used by IAV to increase PGE_2_ and evade antiviral responses remain unknown.

#### Parainfluenza 3 virus

Luczak et al. (1975) showed that addition of PGE_2_ concentrations of 0.1–10 μg/ml to parainfluenza 3 (PIV3) virus infected WISH cells, inhibited the replication of PIV3. They observed that the effect was the most prominent when PGE_2_ was present in the media throughout the replication cycle, compared to PGE_2_ being added before infection and 2 h post-infection. They postulated that PGE_2_ may affect the replication of PIV3 by influencing the growth of WISH cells.

#### Lymphocytic choriomeningitis virus

It has been shown that PGE_2_ can impair the survival and effector functions of T_c_ during chronic lymphocytic choriomeningitis virus (LCMV) infection (Chen J. H. et al., 2015). When PGE_2_ signaling was blocked either directly (deletion of EP2 and EP4) or systemically (deletion of mPGES-1 or COX inhibitors) there was an increase in antigen specific T_c_ numbers and cytokine production. Chen J. H. et al. (2015) also found that the simultaneous inhibition of PGE2 and PD-1 signaling (programmed cell death signaling) during LCMV infection increased the numbers, function and viral control of Tc. They concluded that the comodulation of PGE2 and PD-1 signaling could be a therapeutic avenue in the treatment of certain chronic diseases.

#### Respiratory syncytial virus

Macrophages and DCs from newborns were infected with respiratory syncytial virus (RSV) and showed an increase in IL-10, IL-11, and PGE_2_ generation (Bartz et al., 2002). These authors concluded that PGE_2_ and the cytokines might contribute to the predominance of Th2 being produced by DCs during ongoing RSV infection. This in turn could explain the delayed protective RSV specific immune response. In 2005 Liu and co-workers showed that PGE_2_ is produced by human alveolar type II-like epithelial (A549) cells infected with RSV and that it is required for viral replication. They found that RSV infection induces a time-dependent increase in both COX-2 mRNA expression and protein synthesis and thus leads to the enhanced production of PGE_2_. Furthermore, when PGE_2_ production was inhibited by blocking COX-2 or cPLA_2_ activation, there was a significant reduction in RSV replication. They concluded that the reduction in RSV infection is partly due to a significant inhibition of RNA transcription. Walsh et al. (2016) showed that ibuprofen (a NSAID) can decrease PGE_2_ production during RSV infection in 5 to 6 week-old outbred pre-ruminant bottle-fed Holstein bull calves. The decrease in PGE_2_ production in turn modulated the immune responses and improved clinical outcomes. However, they also found that treatment with ibuprofen did not affect lung histopathology and lead to increased viral shedding. They concluded that RSV should thus be treated with both antivirals and immunodulators.

### Single-stranded RNA-RT viruses

#### Human T-lymphotropic virus type III

Kuno et al. (1986) investigated the direct effects of PGE_2_ on a T-cell line (MT-4 cells) when infected with type III human T-lymphotropic virus (HTLV-III) as well as on HTLV-III continuous-producer cells (Molt-4/HTLV-III cells). They found that PGE_2_ enhanced the production and release of infectious virus in a dose-dependent manner. They concluded that PGE_2_ or its metabolites may play a role in the activation of transcription/translation of HTLV-III integrated genes or in the maturation of infectious viral particles. It is unclear if PGE_2_ plays any role in early infection before the integration of HTLV-III genes.

#### Human immunodeficiency virus

In 1998 Olivier and Tremblay showed that PGE_2_ had a upregulating effect on the long terminal repeat (LTR) gene of human immunodeficiency virus type-l (HIV-l) in Jurkat E6.1 cells. HIV-1 protein expression is driven by HIV-1 LTR. They found that this activation of HIV-1 by PGE_2_ was transduced via both, NF_K_β-dependent and –independent, signaling pathways. It was concluded that the secretion of PGE_2_ by macrophages in response to infection or inflammatory activators could induce signaling pathways that results in the activation of proviral DNA present in T cells latently infected with HIV-1. When PGE_2_ is added to macrophages 24 h prior to infection with HIV-1 the cells show resistance to infection (Thivierge et al., 1998). The increase in PGE_2_ leads to increased cAMP levels which in turn downregulate the expression of CCR5 (coreceptor for HIV-1 entry). This inhibitory effect has also been observed in the replication of both M- tropic HIV-_1BAL_ and HIV-1 in monocyte-derived macrophages (MDM) and monocytic cell line (UI), respectively (Hayes et al., 2002). The inhibition appears to rely on the cAMP/PKA-dependent mechanism and functions at a gene expression level decreasing HIV mRNA. Hayes et al. (2002) demonstrated that the regulation of inhibition by the cAMP/PKA-dependent mechanism is through HIV-1 promoter activity. Women infected with human papillomavirus and HIV-1 showed an increase in cervical COX-2 and elevated systemic PGE_2_ levels (Fitzgerald et al., 2012) which may account for the immunosuppressive effects observed in infected patients. Samikkannu et al. (2014) showed that AA and its metabolites and accompanying enzymes (PGE_2_ and COX-2, respectively) mediate the accelerative effect of cocaine on HIV infection by impairing possible immune functions. PGE_2_ can also act in the late stages of HIV-1's viral cycle (Clemente et al., 2014). Treatment with PGE_2_ increased the activity of Rab1 (molecular switches that regulate membrane traffic), decreased RhoA (associated with cytoskeleton regulation) activity, and subsequently reduced the polymerization of actin. Furthermore, viral assembly platforms enriched with group-specific antigen (Gag) were disrupted. Taken together these results led Clemente et al. (2014) to conclude that PGE_2_ reduces the infectivity of HIV-1 by affecting the spread of virions from cell-to-cell. In addition Zambrano-Zaragoza et al. (2014) suggested that Th17 cells and the Th17/Treg balance could maintain HIV under control and could therefore play a role in the disease progression of AIDS (Brandt et al., 2011). The ratio of Treg/Th17 showed a negative correlation to viral plasma load (Chevalier et al., 2013), although Treg cells correlated positively with viral load before antiviral therapy (He et al., 2012). Antiretroviral treatment normalizes this ratio in HIV patients (Brandt et al., 2011; He et al., 2012). Thus, PGE_2_ appears to have both an inhibitory and stimulatory effect on the replication of HIV-1 depending on specific conditions and could play a role in HIV pathogenicity via the regulation of Th17 cells.

### Double-stranded DNA-RT viruses

#### Hepatitis B virus

The treatment of recurrent hepatitis B virus (HBV) infection after orthotopic liver transplantation with PGE_2_ has had some beneficial effects (Flowers et al., 1994). PGE_2_, given intravenously, followed by oral therapy, arrested or ameliorated recurrent infection in 67% of treated patients. Flowers et al. (1994) speculated that the mechanism of PGE_2_ interference might rely on its ability to stabilize cellular membranes and inhibit HBV binding and replication. Hyman et al. (1999) also showed that PGE_2_ has a beneficial effect on chronic HBV, but no effect on chronic hepatitis C (HCV). Patients were treated with PGE_2_ for 6 months and 47% of patients suffering from chronic HBV showed a decrease in HBV viral parameters [serum HBV DNA and HBeAg (envelope antigen of hepatitis B)]. In 2009, Xie and co-workers found that celecoxib (COX-2 inhibitor) had potent inhibitory effects on the growth of hepatitis B virus X protein positive hepatocellular carcinoma cells (HepG2-X) (Xie et al., 2009). When exogenous PGE_2_ was added to HepG2-X cells the inhibitory effect of celecoxib was slightly overcome. The exact beneficial mechanism of PGE_2_ is still unknown. T helper 17 cells as well as the ratio of Treg/Th17 appear to have a crucial role in the occurrence, development and outcome of HBV (Sun et al., 2012; Xue-Song et al., 2012). Interleukin 17 (cytokine of Th17 cells) has been shown to be indispensable for HBVs antigen (HBsAg)-stimulated differentiation of CD4^+^ cells into Th17 (Yang et al., 2013). Thus, Th17 cells have been show to participate in the pathogenesis of liver damage associated with HBV (Yang et al., 2013). It could, thus, be plausible that levels of PGE_2_ may play a role in the pathogenicity of HBV via Th17 cell differentiation.

## Prostaglandin E_2_ as a potential therapeutic target

The current review highlights the potential of the biosynthetic pathway of PGE_2_ (Figure 1) as a therapeutic target in viral infections. This is possible as PGE_2_ has been shown to play a role in various viral infection, by either having a stimulatory/inhibitory effect on the viral life cycle or host's immune system.

One of the first potential therapies is limiting the amount of AA (or FA that can be converted to AA) that is taken in up in the diet of an individual (Calder, 2005, 2010). This can be done by limiting the amount of n-6 polyunsaturated fatty acids (PUFAs) (vegetable oils, animal sources) and rather ingesting n-3 PUFAs (fish oils, marine sources) which are preferentially converted into either docosahexaenoic acid (DHA) or eicosapentaenoic acid (EPA). They act by replacing AA as an eicosanoid substrate and inhibiting AA metabolism (directly) or altering the effects of inflammatory genes through effects on transcriptional activation (indirectly) (Calder, 2005). Although EPA also gives rise to eicosanoids, these have anti-inflammatory effects in contrast to AA-derived eicosanoids (Calder, 2006).

Both of the COX isoforms can also serve as a potential target as they are directly responsible for the downstream production of PGE_2_. Of the two isozymes, COX-2 is a more attractive target as COX-1 is constitutively expressed and particularly important in gastrointestinal protection (Hawkey, 2001). This means that COX-2 selective drugs are required to regulate the production of PGE_2_. These drugs include coxibs (celecoxib and rofecoxib), etodalac, meloxicam, and nimesulide. Coxibs function by binding to side pockets close to the COX-2 active site, while etodalac, meloxicam and nimesulide bind in the active site (Jackson and Hawkey, 2000). All of these selective inhibitors have some side effects and can only partially inhibit PGE_2_ production.

The next possible therapeutic targets include the PGES (Murakami and Kudo, 2006). Microsomal prostaglandin E synthase-1 is the most promising target as the deletion of mPGES-1 in animal and cells models leads to no severe adverse effects (Chen Y. et al., 2015). There are no known mPGES-1 inhibitors due to the fact that compounds are not stable or effective in cellular assays or animal models. The search for an appropriate inhibitor is further complicated by specific differences in rodent and human models as they differ in key amino acid residues of mpGES-1 active site. Inhibitors also need to overcome the challenge of the complex binding mechanisms of both cofactors and substrates. Although all of these enzymes are potential therapeutic targets, care should be taken in the way these targets are inhibited as several of the downstream products of the of the PGE_2_ biosynthetic pathway are required for normal physiological functioning. The effect of the target should also take into account the effect that lowering/increasing PGE_2_ level may have on preexisting autoimmune conditions or in causing such conditions.

## Conclusions

In response to viral infections the host elicits a defense by activating inflammation and immunity. The activation of inflammation by viral infections leads to the production of PGE_2_ which has a modulatory role in immunity and other roles in normal cell physiology. In this review, viruses from specific classes including, double-stranded DNA viruses (HSV, CMV, EBV, MAV-1), double-stranded RNA viruses (RV), (+) single-stranded RNA viruses (CVB3, EV71, PSaV), (−) single-stranded RNA viruses (VSV, IVA, PIV3, LCMV, RSV), single-stranded RNA-RT viruses (HTLV-III and HIV-l) and double-stranded DNA-RT viruses (HBV) and their interaction with PGE_2_ were reviewed. PGE_2_ had either an inhibitory, stimulatory or in some cases a dual role in there viral replication cycles. The stimulation of viral pathogenicity (HSV, CMV, EBV, RV, CVB3, EV71, PSaV, VSV, LCM, RSV, and HTLV-III) occurred mainly by affecting the host immunity, viral transcription/translation, and/or viral replication, while the inhibition (PIV3 and HBV) affected viral replication itself. In the case of both IAV and HIV-1, PGE_2_ was shown to have both a stimulatory and inhibitory effect. Both these viral stimulations were dependent on immune suppression and gene expression, while the inhibitory effects were dependent on IL-27 expression (IAV) and inhibition of spreading (HIV-1). In many of the viral infections the effect of PGE_2_ was negated when inhibitors of PGE_2_ synthesis was added, but the effects were overcome by the addition of exogenous PGE_2_. Interestingly, it appears that Th17 cells have a regulatory role in retrovirus infections. In turn Th17 cells are regulated by PGE_2_ which might play an indirect role in these viral infections. There are a number of possible therapeutic targets in the PGE_2_ biosynthetic pathway, although more research into their effects and modes of actions are required. This paper suggest that it may be possible to design therapeutic strategies to target selective pathways in an effort to attenuate inflammation associated with virus infection.

When looking into the majority of studies included in this review, the effect that PGE_2_ has in viral infections seem to have been a coincidental discovery and not the primary objective of these studies. In some cases the discovery was noted, but not further investigated. There is, therefore, a major gap in our knowledge of the exactly role of PGE_2_ in viral infections. These gaps include, determining the exact signaling pathways that viruses might use to induce PGE_2_, the role that PGE_2_ can play in the induction of autoimmunity and clarifying the dependence of viral infections of PGE_2_. The underlying mechanisms should be further investigated to determine if viruses require PGE_2_ to enhance their pathogenicity or require PGE_2_ for optimal viral replication. Furthermore, light should be shed on the possible effect that the interplay between TLRs, PGE_2_ and its receptors may have on viral infections. Taken together these mechanisms could also shed light on other host-pathogen interactions and may facilitate selection or development of the more optimal therapies against viral infections.

## Author contributions

WS compiled the information, co-wrote the manuscript, and approved the final version submitted. HO and CP provided scholarly input in placing the literature into context, edited the manuscript, and approved the final version submitted.

## Funding

The study was funded through the South African National Research Foundation (Grant No: 103395 to HO).

### Conflict of interest statement

The authors declare that the research was conducted in the absence of any commercial or financial relationships that could be construed as a potential conflict of interest. The reviewer MR and handling Editor declared their shared affiliation, and the handling Editor states that the process nevertheless met the standards of a fair and objective review.
